# The Ties That Bind: University Nostalgia Fosters Relational and Collective University Engagement

**DOI:** 10.3389/fpsyg.2020.580731

**Published:** 2021-02-01

**Authors:** Jeffrey D. Green, Athena H. Cairo, Tim Wildschut, Constantine Sedikides

**Affiliations:** ^1^Department of Psychology, Virginia Commonwealth University, Richmond, VA, United States; ^2^Centre for Research on Self and Identity, Department of Psychology, University of Southampton, Southampton, United Kingdom

**Keywords:** nostalgia, volunteering, donation, social connectedness, subjective well-being, reunions, identity

## Abstract

Does nostalgia for one’s time at university predict current intentions to engage with the university? In Study 1, United States participants’ nostalgia for their university experience (university nostalgia) at a southern public university predicted stronger intentions to socialize with fellow alumni, attend a future reunion, volunteer for their university, and donate money to their university. Study 2 replicated these findings with alumni from a northeastern private university, and extended them by finding that the links between university nostalgia and university engagement emerged even when controlling for the positivity of university experience. In both studies, feelings of university belonging mediated most of the associations between university nostalgia and university engagement. In Study 2, the positivity of the university experience moderated the relation between university nostalgia and two indices of university engagement. Specifically, university nostalgia was more strongly associated with intentions to attend a reunion and donate money among those who had a relatively negative university experience. Nostalgia for one’s university past predicts future engagement with the university as well as its members.

## Introduction

Until the late 20th century, nostalgia—a bittersweet emotion associated with somewhat rosy recollections of the past—was characterized in both historical and empirical works as a mental affliction akin to homesickness (Sedikides et al., 2004). However, in recent decades, psychological research has distinguished distress-related emotions—such as homesickness and separation anxiety—from the wistful, warm, and sentimental nature of nostalgia. For example, participants’ descriptions of nostalgic memories are predominantly characterized by positive affect and feelings of connectedness with significant others (Wildschut et al., 2006). In addition, nostalgia acts as a psychological buffer or antidote against social disconnection and negative affect. For example, nostalgic memories are more likely to be evoked while experiencing negative affect (Wildschut et al., 2006) or when feeling lonely (Zhou et al., 2008). When one endures aversive states, nostalgic memories act as a repository of positive affect, self-regard, and social connectedness (Wildschut et al., 2011; Wildschut and Sedikides, 2020). In particular, nostalgic memories reset psychological equilibrium through enhancing symbolic connections with others (Sedikides et al., 2008a; Abeyta et al., 2015).

Nostalgia motivates action (Sedikides and Wildschut, 2020). It is an approach-oriented emotion (Van Tilburg et al., 2018) that can increase inspiration (Stephan et al., 2015), risk-taking (Zou et al., 2019), and the pursuit of important life goals (Sedikides et al., 2018). Nostalgia can fuel behavioral intentions and actual behavior in areas as varied as purchasing food with nostalgic labels (Zhou et al., 2019) or engaging with favorite sports teams, such as visiting a sports town (Chang et al., 2019).

Most germane to our work, nostalgic recollections satisfy the need for social connection not only by buffering against loneliness, but also by motivating favorable intentions and behaviors toward others. Nostalgia strengthens intentions to support one’s ingroup (Wildschut et al., 2014) and increases charitable donations (Zhou et al., 2012). Furthermore, when individuals become nostalgic for their ingroup, they bestow unique benefits on it, including tangible monetary sacrifices to support its members (Wildschut et al., 2014). We sought to investigate the link between nostalgia at the collective level and social orientations as well behaviors toward an important social group: one’s university alma mater.

University nostalgia is the wistful longing for and recollection of the formative university years. Young adulthood is a particularly rich source of nostalgia (Rubin and Schulkind, 1997; Batcho, 1998; Wildschut et al., 2006): individuals display heightened recall from this life era in particular (i.e., the reminiscence bump; Koppel and Berntsen, 2015), and they experience nostalgia for their adolescence *via* music, photos, time with old friends, and more (Barrett et al., 2010; Sedikides et al., 2015b). College is replete with the cherished social connections and momentous events that form the wellspring of nostalgic memories (Holak and Havlena, 1992; Wildschut et al., 2006; Madoglou et al., 2017). The university experience is for most a time to explore novel ideas, form numerous close relationships, and try new activities, all of which can be fodder for future nostalgia *via* “anticipated nostalgia” (Cheung et al., 2020) and savoring (Biskas et al., 2019). United States universities in particular may foster powerful memories for individuals who move away to college, live in a quintessential college town, or embrace an active college sports culture. University experiences vary, of course, for individuals, universities, and cultures, but university nostalgia likely is potent across a range of cultures due to its unique characteristics and the life stage involved (Rathbone et al., 2017).

## Collective Nostalgia, Group Collectivism, and Relational Collectivism

A tripartite view of the self—individual, relational, and collective—has been a generative framework for empirical research (Sedikides and Brewer, 2001; Sedikides et al., 2013), including self-relevant emotions. The individual self, the unique set of characteristics (e.g., traits, history, and worldview) that sets one individual identity apart from others, has attracted the bulk of attention empirically (in part because it tends to be prioritized over the other two; Gaertner et al., 2012; Nehrlich et al., 2019), particularly regarding nostalgia (i.e., personal nostalgia; Sedikides et al., 2015b). The relational self refers to identification with dyadic relationships and close-knit, interdependent groups like families. It includes relationship-specific roles, memories, traits, and goals. The collective self refers to identification with larger social groups and categories (Sedikides et al., 2013). It includes emphasis on a shared history, usually one that differentiates the ingroup from relevant outgroups [e.g., national nostalgia; (Smeekes et al., 2018)]. Relational nostalgia (Hepper et al., 2012b; Mallory et al., 2018) and collective nostalgia (Wildschut et al., 2014; Smeekes, 2015) have only recently been explored empirically.

Intergroup emotions theory (IET; Mackie and Smith, 1998, 2018) proposes that intergroup emotions are an inevitable byproduct of individuals considering their group identities. Membership in important groups elicits group-level counterparts to emotions that people experience as individuals. Group norms, practices, and history influence the experience and regulation of a host of intergroup emotions, including nostalgia. Recent work defined *collective nostalgia* as “the nostalgic reverie that is contingent upon thinking of oneself in terms of a particular social identity or as a member of a particular group…and concerns events or objects related to it” (Wildschut et al., 2014, p. 845), and established collective nostalgia as an intergroup emotion according to the principal tenets of IET. Collective nostalgia can be distinguished from its individual-level counterpart (i.e., personal nostalgia; Sedikides and Wildschut, 2019; Abakoumkin et al., 2020)and, and collective nostalgia regulates and directs attitudes and behavior toward the collective. For example, individuals who experienced collective nostalgia by reflecting on a shared memory felt more positively about their group, reported stronger motivation to approach ingroup members, and were more willing to make a financial sacrifice to punish anti-ingroup behavior (Wildschut et al., 2014).

Virtually all collective nostalgia research has centered on national nostalgia (Smeekes et al., 2014; Smeekes, 2015; Martinovic et al., 2017). Smeekes (2015) hypothesized that national nostalgia renders salient an “essentialist national ingroup prototype” (p. 64) that limits who is considered part of the national identity. Greater national nostalgia has been associated with a stronger ethnic national identity (Smeekes, 2015) and more negative attitudes toward outgroups (Smeekes et al., 2014; Smeekes, 2015). However, collective nostalgia also can spark greater ingroup loyalty, such as a preference for ingroup (e.g., domestic vs. foreign) consumer products (Dimitriadou et al., 2019).

Recent work on collectivism has distinguished between *group collectivism* and *relational collectivism* to address conceptual and empirical problems in the literature on individualism and collectivism (Brewer and Chen, 2007). Group collectivism refers to social identification with larger, abstract, and depersonalized social groups like nationality or race, and is characterized by a sense of group loyalty and conformity to group norms. Relational collectivism refers to social identification at the small-network level (e.g., family and friends), and is characterized by concerns with harmony, reciprocity, cohesion, and responsiveness to others. Past research on collective nostalgia has not distinguished between relational collectivism and group collectivism [but see Abakoumkin et al. (2020)], and has focused almost exclusively on group collectivism, primarily national nostalgia. We propose to fill a gap in the literature by studying a form of collective nostalgia at the relational level: university nostalgia. Might nostalgia for one’s university days be associated with social orientations and behavioral intentions in the present and future?

Two prior studies (Wildschut et al., 2014, Studies 1 and 2) examined university nostalgia, and found that those who recalled a shared university memory (relative to a personal memory) felt more positively about their fellow alumni. Those experiencing university nostalgia also reported a stronger approach orientation and stronger behavioral intentions to invest time supporting their university by participating in a publicity campaign. We extended this past work in several ways. We examined intentions to engage with the university community at both the relational (i.e., dyadic) and collective levels. We assessed intentions to volunteer to serve the university in various ways (while controlling for past volunteering). We also assessed intentions to donate money to the university (while controlling for past donations as well as income level). In addition, we tested whether university nostalgia would influence university engagement at the dyadic level. Would university nostalgia be linked to intentions to connect with fellow alumni? We included two measures of dyadic engagement: intention to attend a future reunion and time spent with fellow alums (while controlling for past engagement).

## Proposed Mediators: Social Connectedness, Identification, and Self-Continuity

How might university nostalgia be linked to university engagement? That is, what is it about feeling nostalgic for university life that might explain intentions to be more engaged with the university and fellow community members? Past research on nostalgia suggests three promising potential mediators: social connectedness, group identification, and self-continuity.

### Social Connectedness

Nostalgic memories are social (Wildschut et al., 2006). Humans have a fundamental need to belong (Baumeister and Leary, 1995). Individuals go to great lengths to establish and maintain close relationships and social networks, and loneliness and social exclusion or ostracism are subjectively distressing, as well as interpersonally disruptive (Twenge et al., 2002; Baumeister et al., 2005). Individuals who experience nostalgia feel more loved and connected (Reid et al., 2015), more securely attached (Wildschut et al., 2010), and more socially supported (Zhou et al., 2008). They express stronger social approach goals and even sit closer to strangers (Stephan et al., 2014). Lonely individuals are more likely to bring to mind nostalgic memories, which reduce their loneliness *via* increased feelings of social support (Zhou et al., 2008). In addition to this regulatory function, social connectedness mediates downstream effects of nostalgia. Nostalgic (vs. ordinary) memories enhance optimism (Cheung et al., 2013) and inspiration (Stephan et al., 2015) by way of social connectedness.

### Group Identification

Prior research suggests that collective nostalgia culminates in positive ingroup outcomes by increasing group identification. Wildschut et al. (2014) showed that collective nostalgia (compared to a control group) strengthened participants’ willingness to volunteer their time for a university publicity campaign, and this effect was mediated by ingroup identification (indexed by the Collective Self-Esteem Scale; Luthanen and Crocker, 1992). Similarly, Dimitriadou et al. (2019) found that collective nostalgia augmented participants’ preference for ingroup consumer products (i.e., domestic country bias) *via* increased group identification (also indexed by the Collective Self-Esteem Scale). Thus, we propose that group identification may serve as a mediator between university nostalgia and group engagement. At the collective level, group identification and social connectedness may overlap considerably. Group identification by definition entails seeing oneself as part of the collective, and sharing characteristics and goals with other group members. Accordingly, individuals who identify strongly with a group will manifest a stronger sense of social connectedness to other group members.

### Self-Continuity

Self-continuity refers to a sense of coherence and connection of one’s self across time (Sedikides et al., 2015a). This enhanced connection and similarity of the past self and present self can pertain to individual as well as to collective selves (Sedikides et al., 2008b). Nostalgia, induced in different ways, has been shown to enhance self-continuity. For example, individuals who experienced greater nostalgia by smelling familiar scents (Reid et al., 2015), recalling nostalgic autobiographical memories (Abakoumkin et al., 2019), or reading the lyrics to nostalgic songs (Sedikides et al., 2016) felt more continuity in their lives. Self-continuity, in turn, is associated with positive outcomes, such as eudaimonic wellbeing (Sedikides et al., 2016) and meaning in life (Van Tilburg et al., 2019). We reasoned that self-continuity would mediate the effect of university nostalgia on positive engagement with the university and its members.

## Overview and Hypotheses

We conducted two studies, one with graduates of a public university in the southern United States, and one with graduates of a private northeastern university in the northeastern United States. We hypothesized that university nostalgia would be associated with university engagement at the relational and collective levels. To be specific, we predicted that greater university nostalgia would be associated with greater engagement with fellow alumni (relational outcomes) and greater engagement with the university (collective outcomes) (Hypothesis 1). We also hypothesized that these links would be mediated by social connectedness, identification with the university, and self-continuity (Hypothesis 2).

## Study 1

### Method

#### Participants

Participants were 310 alumni (229 women, 62 men, one other gender, and 18 refused to answer) from a large, public United States university, who completed the survey online for a chance to win one of three gift cards. Participants’ age ranged from 21–79 years (*M* = 40.95, *SD* = 12.89). They were 80% White, 6.1% African American/Black, 6.7% East/South Asian, and 7.2% Multiracial/Other. Participants’ yearly household income ranged from $0–$1,000,000, with a median income of $100,000 (*M* = $138,498, *SD* = $117,428).

#### Procedure and Measures

After indicating their year of graduation, participants responded to the survey measures and behavioral engagement items reported below. At the end of the session, they were given the opportunity to enter a gift card raffle as compensation.

### University Nostalgia

To measure university nostalgia, we used a university-specific version of the Southampton Nostalgia Scale (Sedikides et al., 2015b; Wildschut and Sedikides, 2021), which assesses both frequency and importance of nostalgic engagement. We adjusted slightly the wording for nostalgia to refer to participants’ alma matter. Sample items include: “How often do you experience nostalgia about X University” and “How valuable to you is feeling nostalgic for X University?” (1 = *not at all*, 7 = *very much*). Cronbach’s alpha was 0.95.

### Connectedness to University Community

We measured participants’ connectedness with their university community with three items used in previous research to assess social connectedness (Wildschut et al., 2006; Hepper et al., 2012a). The items were: “I feel like I’m a part of the X University community,” “I feel that I am a part of the greater X University ‘family’,” and “I still maintain strong ties with friends from X University” (1 = *strongly disagree*, 6 = *strongly agree*). Cronbach’s alpha was.82.

### Identification With University Community

We measured participants’ identification with their university community with three items adapted from the Social Identification Scale (Tarrant et al., 2004). We selected three items from the original scale in order to keep the online study as concise as possible. The items were: “I identify strongly with X University,” “Being a X University graduate is a significant part of my identity,” and “Feeling more identified with X University is important to me” (1 = *strongly disagree*, 6 = *strongly agree*). Cronbach’s alpha was 0.91.

### Self-Continuity

We measured self-continuity with participants’ university-aged selves with the 4-item State Self-Continuity Scale (Sedikides et al., 2015a). We modified slightly the items to refer to participants’ university-aged selves. Sample items include: “I feel connected with my past at X University” and “I feel that there is continuity between my life at X University and my current life” (1 = *strongly disagree*, 6 = *strongly agree*). Cronbach’s alpha was 0.81.

### Socializing With Other Alumni

Participants indicated their interest in socializing with other alumni by responding to the item: “How much do you plan to socialize with other alumni from your university in the next year?” (1 = *not at all*, 7 = *all the time*). Participants also indicated socializing tendencies by responding to the item: “How often do you socialize with other alumni from your university?” (1 = *never/almost never*, 7 = *once a week or more*).

### Volunteering

Participants reported their willingness to volunteer for their alma mater by responding to the items: “If asked by someone from University X, would you be willing to volunteer for the university in the near future?” (*yes/no* response) and “If so, how many hours would you be willing to volunteer?” (numeric response). Participants also reported prior volunteering by responding to the items: “Have you ever volunteered for your alma mater after you graduated (for example, recruitment events or service projects)?” (*yes/no* response) and “If so, approximately how many total hours have you volunteered for your alma mater?” (numeric response).

### Reunion Interest

Participants indicated their interest in attending an upcoming class reunion by responding to the item: “How much interest do you have in attending the next reunion?” (1 = *no interest at all*, 7 = *extremely interested*). Participants also reported prior reunion attendance by responding to the item: “How many official reunions have you attended at X University?” on a numeric scale. Lastly, participants stated the total number of their class reunions held (We needed this information to calculate a ratio of reunion attendance).

### Charitable Donations

Participants responded to several measures relating to charitable donations to their alma mater. They reported willingness to donate to the university by responding to the items: “If asked by someone from University X, would you be willing to donate to them in the near future?” (*yes/no* response) and “If so, how much do you think you would be willing to donate?” (numeric response). They also reported whether they had ever donated to their alma mater in the past (*yes/no* response) as well as the largest gift amount, average gift amount, and number of years they had previously donated (numeric responses).

### Demographics

Participants indicated their gender, ethnicity, age, household income, where they currently lived, whether they had children currently attending their alma mater, whether their significant other was also an alum, and the number of university friends with whom they regularly kept in contact.

### Results

#### Analysis Strategy and Descriptive Statistics

We checked all continuous dependent variables for linearity and normality of residuals. We log-transformed the variables of income, planned and average yearly hours of volunteering, and planned and average donation amount, as they were positively skewed and did not meet assumptions for normality. Following these transformations, all variables met assumptions for linear regression. Additionally, we screened data for inattentive responding *via* three questions (e.g., *Choose “very strongly” for this answer*). We removed seven participants for missing at least two attention check questions.

Table 1 displays descriptive statistics of Study 1 variables, and Table 2 displays bivariate correlations among Study 1 variables. Given that the connectedness and identification measures were highly correlated [*r*(308) = 0.78, *p* < 0.001], we combined them by averaging all six items into a single measure that we label *university belonging*. Cronbach’s alpha for this composite measure was 0.91.

**TABLE 1:** Descriptive statistics of study 1 variables.

Variables	Mean/%	SD
1. University Nostalgia	4.77	1.39
2. Graduation year	1999	13.08
3. Income (thousands)	$138.5	$117.4
4. Connectedness	5.22	1.50
5. Identification	5.32	1.54
6. Belongingness	5.67	1.43
7. Self-continuity	4.55	1.00
8. Past socialization	4.55	1.91
9. Plans to socialize	4.58	1.83
10. Past volunteerism (hours)	25.10	51.11
11. Willingness to volunteer (yes/no)	70.0%	
12. Willingness to volunteer (hours)	4.17	15.74
13. Past reunion attendance (number of reunions)	0.27	0.71
14. Future reunion interest	3.69	2.02
15. Past donation - avg. gift (dollars)	$172	$466
16. Past donation - largest gift (dollars)	$495	$1892
17. Willingness to donate (yes/no)	58.7%	
18. Willingness to donate (dollars)	$536	$5,746

*Median income = $100,000. University Nostalgia and Reunion Interest variables are on a 1 *(low*) to 7 (*high*) rating scale. Connectedness, identification, self-continuity, and past/planned socialization variables are measured on a 1 (*low*) to 6 (*high*) rating scale.*

**TABLE 2:** Descriptive statistics and bivariate correlations among study 1 variables (*N* = 310).

Variables	1	2	3	4	5	6	7	8	9	10	11	12	13	14	15	16	17	18
1. University Nostalgia	–																	
2. Graduation year	−0.15*	–																
3. Income	–0.03	0.10	–															
4. Connectedness	0.63***	–0.04	–0.08	–														
5. Identification	0.76***	−0.13*	−0.15*	0.77***	–													
6. Belongingness	0.74***	–0.10	–0.12	0.94***	0.94***	–												
7. Self-continuity	0.49***	–0.04	−0.13*	0.57***	0.49***	0.56***	–											
8. Past socialization	0.26***	0.02	0.05	−0.12*	−0.18**	−0.16**	–0.11	–										
9. Plans to socialize	0.38	0.09	0.12	–0.09	–0.05	–0.07	−0.12*	–0.05	–									
10. Past volunteerism	0.09	–0.15	–0.08	0.21*	0.15	0.19	0.15	0.00	−0.24*	–								
11. Future volunteerism (y/n)	0.21**	0.05	–0.02	0.01	0.00	0.00	0.08	0.03	0.22***	–0.16	–							
12. Future volunteerism amount	0.20*	–0.02	0.02	0.00	–0.02	–0.01	0.03	0.02	0.24***	–0.13	0.82***	–						
13. Past reunion attend.	0.11	–0.12	–0.09	0.17*	0.16	0.17*	0.21**	–0.08	–0.03	–0.11	0.08	0.15	–					
14. Future reunion interest	0.46**	–0.03	0.03	0.03	–0.04	–0.01	0.07	–0.06	0.42***	–0.18	0.34***	0.35***	0.11	–				
15. Past average gift amount^†^	0.05	0.02	–0.05	0.07	0.04	0.06	0.08	0.01	–0.09	0.08	0.01	–0.03	0.24*	–0.01	–			
16. Past largest gift amount^†^	0.09	0.06	0.02	0.10	0.07	0.09	0.11	0.08	–0.09	0.14	–0.05	–0.06	0.19	0.00	0.75***	–		
17. Future willing to donate (y/n)	0.30***	−0.18**	–0.08	0.28***	0.30***	0.31***	0.15*	0.04	–0.07	0.06	–0.02	–0.03	0.07	–0.01	0.10	0.12	–	
18. Future willing donation amount^†^	0.25***	−0.15**	–0.03	0.28***	0.29***	0.30***	0.15**	0.05	–0.03	0.01	0.00	–0.01	0.13	0.01	0.09	0.08	0.86***	–

*p < 0.05, **p < 0.01, ***p < 0.001, and † variable log-transformed due to strong positive skewness.Univ, university; Attend., attendance.

To assess the relation between university nostalgia and alumni engagement outcomes, we ran hierarchical regressions with covariates (prior engagement and year of graduation in all models, and log-transformed income for all models (except subjective well-being and socializing interest) in step 1, and university nostalgia in step 2. These models assessed the association of university nostalgia with alumni engagement, above and beyond graduation year, income, and prior engagement in the behavior. We controlled for graduation year to account for the effect of time on participants’ level of nostalgia. We controlled for income to account for the possibility that those with higher incomes have more resources to take time off for volunteering or visiting other alumni, as well as donating to their alma mater. We controlled for prior engagement, as it has been shown to be a strong predictor of future engagement in related behavior (Ouellette and Wood, 1998). We summarize these regression models in Table 3.

**TABLE 3:** Regression models for study 1 alumni engagement outcomes.

Dependent variable (Continuous)	Predictor	Step 1						Step 2					
		β	*p(*β*)*	*F*	*df*	*p*(*F*)	*R^2^_*adj*_*	β	*p(*β*)*	*F*	*df*	*p*(*F*)	*R^2^_*adj*_*
Socializing with other alumni	Graduation year Past socialization University Nostalgia	−0.10 0.74	0.028 <0.001	149.48	2, 288	<0.001	0.51	−0.03 0.66 0.23	0.474 <0.001 <0.001	118.29	3, 287	<0.001	0.55
Volunteering (hours)	Graduation year Past volunteering amount Income University Nostalgia	−0.26 0.05 −0.14	0.032 0.660 0.249	1.82	3, 71	0.152	0.03	−0.22 0.02 −0.15 0.46	0.048 0.853 0.160 <0.001	6.79	4, 70	<0.001	0.28
Reunion interest	Graduation year Past reunion attendance Income University Nostalgia	−0.05 0.28 −0.07	0.480 <0.001 0.294	6.15	3, 205	<0.001	0.07	0.01 0.23 −0.04 0.43	0.816 <0.001 0.476 <0.001	17.82	4, 204	<0.001	0.24
Donation (amount)	Graduation year Past donation average amount Income University Nostalgia	−0.06 0.34 0.19	0.430 <0.001 0.010	14.22	3, 166	<0.001	0.19	0.02 0.31 0.22 0.26	0.820 <0.001 0.002 <0.001	15.23	4, 165	<0.001	0.25

		***OR***	***p(OR)***	**Model χ2**	***df***	***p*(χ2)**	***Nagelkerke R^2^***	***OR***	***p(OR)***	**Model χ2**	***df***	***p*(χ2)**	***Nagelkerke R^2^***

Volunteering (y/n)	Graduation year Past volunteering amount Income University Nostalgia	0.88 1.00 0.84	0.286 0.929 0.160	2.38	3	0.498	0.06	0.98 0.99 0.99 4.45	0.548 0.391 0.169 <0.001	24.81	4	<0.001	0.52
Donation (y/n)	Graduation year Past donation frequency Income University Nostalgia	1.01 1.00 1.00	0.719 0.406 0.018	8.66	3	0.03	0.07	1.00 1.00 1.01 1.59	0.940 0.490 0.008 0.001	21.65	4	<0.001	0.17

After establishing the relation between nostalgia and engagement, we assessed the role of potential mediators: connectedness to the college community, identification with the college community, and self-continuity with participants’ college-aged selves. For all mediation analyses, we controlled for graduation year and past engagement in alumni behavior. We also controlled for income in the cases of volunteering, reunion interest, and alumni donations. We conducted mediation analyses with Hayes’ PROCESS macro v3.4 (Hayes, 2018). We calculated bootstrapped confidence intervals of indirect effects using 5,000 re-samplings. For indirect effects of odds ratios (OR), we considered confidence intervals including 1.0 as non-significant. For indirect effects of continuous variables (beta coefficients), we considered confidence intervals including 0 as non-significant.

#### Socializing With Other Alumni

As shown in Table 3, university nostalgia predicted interest in socializing with other alumni, β = 0.23, *p* < 0.001, *R*^2^Δ = 0.04. First, we conducted simple mediation analyses to assess whether university belonging or self-continuity accounted for the relation between nostalgia and socializing with other alumni. Assessed separately, we found a significant indirect effect (denoted as *ab*) *via* university belonging [*ab* = 0.29., 95% CI = (0.16,0.41)]. Self-continuity did not significantly mediate the relation between university nostalgia and socializing [*ab* = 0.03, 95% CI = (−0.01,0.08)]. When we tested both mediators in a parallel mediation model, university belonging uniquely accounted for the relation between university nostalgia and socializing [*ab* = 0.13., 95% CI = (0.02,0.34)]. Self-continuity did not mediate the relation [*ab* = 0.02., 95% CI = (−0.03,0.07)].

### Volunteering

The majority of respondents indicated they would be interested in volunteering for the university if asked to do so (Table 1). As shown in Table 3, we found in a logistic regression that university nostalgia predicted greater likelihood of being willing to volunteer for the alma mater over and above graduation date, income, and previous frequency of volunteering, *B* = 1.49, OR = 4.45, *p* < 0.001. University nostalgia also predicted willingness to volunteer a greater number of hours (log transformed), β = 0.46, *p* < 0.001, and *R*^2^Δ = 0.25.

Assessed in separate simple mediation models, university belonging significantly mediated the relation between nostalgia and willingness to volunteer for the alma mater [*ab* = 1.33, 95% CI = (1.01, 1.86)]. Self-continuity did not mediate the relation in a simple mediation [*ab* = 0.94, 95% CI = (0.78, 1.09)]. When we tested both mediators in a parallel mediation model, university belonging again was a significant mediator of the relation between nostalgia and willingness to volunteer [*ab* = 1.55, 95% CI = (1.01, 2.66)]. Self-continuity did not mediate the relation [*ab* = 0.94., 95% CI = (0.78, 1.09)].

We next examined mediation of the relation between nostalgia and the log-transformed number of hours participants were willing to volunteer. Assessed in separate simple mediation models, neither university belonging [*ab* = 0.09, 95% CI = (−0.01,0.19)] nor self-continuity [*ab* = 0.003, 95% CI = (−0.05,0.05)] significantly mediated the relation between nostalgia and number of hours volunteered for the alma mater. When both mediators were tested in a parallel mediation model, neither university belonging [*ab* = 0.10, 95% CI = (−0.01,0.08)] nor self-continuity [*ab* = −0.01, 95% CI = (−0.06,0.04)] uniquely mediated the relation.

### Reunion Interest

Greater university nostalgia predicted increased interest in attending an upcoming university class reunion, β = 0.46, *p* < 0.001, and *R*^2^Δ = 0.21. Assessed in separate simple mediation models, both university belonging [*ab* = 0.18, 95% CI = (0.02,0.35)] and self-continuity [*ab* = 0.07, 95% CI = (0.01,0.16)] significantly mediated the relation between nostalgia and interest in attending the next class reunion. When we tested both mediators in a parallel mediation model, neither university belonging [*ab* = 0.14, 95% CI = (−0.03,0.31)] nor self-continuity [*ab* = 0.06, 95% CI = (−0.02,0.14)] uniquely accounted for the relation.

### Charitable Donations

Slightly over half of respondents indicated they would be willing to donate some amount to the university if asked to do so (Table 1). University nostalgia predicted greater willingness to donate to the alma mater, *B* = 0.43, OR = 1.53, *p* < 0.001. Participants higher in university nostalgia indicated willingness to donate a greater amount to their alma mater (log-transformed), β = 0.26, *p* = 0.001, *R*^2^Δ = 0.06.

We first examined mediation of the relation between university nostalgia and willingness to donate to the alma mater. In simple mediation models, we found no significant indirect effects of either university belonging [*ab* = 0.15, 95% CI = (−0.10,0.43)] or self-continuity [*ab* = 0.04, 95% CI = (−0.09,0.17)]. When we tested both mediators in a parallel mediation model, neither university belonging [*ab* = 0.14, 95% CI = (−0.13,0.16)] nor self-continuity [*ab* = 0.02, 95% CI =(−0.13,0.16)] mediated the relation.

Next, we examined mediation of the relation between university nostalgia and the log-transformed amount that participants were planning to donate. In a simple mediation model, university belonging was a significant mediator [*ab* = 0.15, 95% CI = (0.003,0.30)]. Self-continuity did not significantly mediate the relation [*ab* = 0.05, 95% CI = (−0.03,0.13)]. When we tested both mediators in a parallel mediation model, neither university belonging [*ab* = 0.12, 95% CI = (−0.06,0.11)] nor self-continuity [*ab* = 0.03, 95% CI = (−0.06,0.11)] uniquely mediated the relation.

### Discussion

Study 1 tested hypotheses concerning the link between university nostalgia and intentions to engage with one’s alma mater. Graduates higher in university nostalgia were more interested in socializing with fellow graduates, willing to volunteer for their alma mater, interested in attending a future reunion, and willing to donate money to the university, supporting Hypothesis 1. Importantly, these links were robust, remaining significant even when controlling for graduation year, relevant past engagement, and income. People who feel more nostalgic for their past at university intend in the future to be more engaged with the university, as well as with fellow alumni. In simple mediation analyses, university belonging mediated the relation between university nostalgia and all measures of university engagement, except willingness to donate money. In parallel mediation analyses (with self-continuity), university belonging remained a significant mediator of university nostalgia’s relation with interest in socializing and willingness to volunteer. These findings provide partial support for Hypothesis 2.

These findings expand on prior university nostalgia research (Wildschut et al., 2014), underscoring the link between nostalgia on the one hand and approach orientation as well as social engagement on the other (Sedikides and Wildschut, 2019, 2020). More broadly, the findings expand understanding of collective nostalgia and behavioral intentions toward the collective. Although these findings are correlational, prior experimental work has established that inducing collective nostalgia can energize positive behavioral intentions directed toward the group (Wildschut et al., 2014).

Study 1 had some limitations, which Study 2 intended to address. The positivity of past university experiences likely correlates both with university nostalgia and the intent to engage with one’s university. Hence, it is important to examine whether the links between university nostalgia and university engagement are unique and remain significant even when controlling for positivity of past university experiences. Second, a potential limitation of the reunion-attendance measure in Study 1 is that reunions only occur once or twice in a decade at most, and attendance could be subject to many extraneous influences (e.g., scheduling conflicts). However, choosing to visit one’s alma mater is not subject to these constraints. Thus, we added a question about non-reunion visits to the university to improve this measure of university engagement.

A third potential weakness is that we did not assess the full range of mediators suggested by the literature. Study 1 examined two mediators, one of which (i.e., self-continuity) played a negligible role. In Study 2, we therefore examined the role of an additional mediator: meaning in life. Nostalgia serves existential functions, such as buffering individuals from the anxiety associated with thinking about their own death (Juhl et al., 2010). Of particular relevance, nostalgia instills a greater sense of meaning in life (Routledge et al., 2011; Reid et al., 2015) and protects individuals from existential threats (Sedikides and Wildschut, 2018). Nostalgia for close others and personally important events helps infuse the present with purpose and significance (Van Tilburg et al., 2019). Furthermore, meaning in life mediates the influence of nostalgia on subjective vitality and intentions to pursue one’s important goals (Routledge et al., 2008; Sedikides et al., 2018). On this basis, we hypothesized that meaning in life would mediate the relation between university nostalgia and university engagement.

Finally, Study 1 focused on the collective level, and so did not assess individual-level outcome. In Study 2, we added subjective well-being to extend the range of outcome variables. Personal nostalgia conduces to subjective well-being (Sedikides et al., 2016; Hepper et al., 2020), and we examined whether university nostalgia is linked to similar subjective well-being benefits. We operationalized subjective well-being as feelings of vitality (Ryan and Frederick, 1997).

## Study 2

Study 1 provided preliminary evidence that university nostalgia is associated with stronger engagement with the university and fellow alumni. It further offered suggestive support for the proposed mediating role of university belonging, but not the role of self-continuity. These findings, however, are in need of replication and elaboration. We pursued these objectives in Study 2. The key objectives were to replicate Study 1 and extend it to address issues we mentioned above such as measuring non-reunion visits, testing meaning in life as a mediator, and assessing subjective well-being (vitality).

In addition, we addressed the role of past university experiences as a control variable. Moreover, we explored whether the positivity of past university experiences would moderate the links of university nostalgia with indices of subjective well-being and university engagement. One possibility is that these links are stronger for individuals who had many positive experiences at university, as they might have a larger store of nostalgic memories that they could seek to recreate *via* current university engagement. Another, more intriguing, possibility is that these links are stronger for individuals who had a relatively negative overall university experience, as the few nostalgic memories that they do cherish assuage their overall negative experience, thereby protecting and sustaining subjective well-being and engagement.

### Method

#### Participants

One hundred and sixty-one alumni of a private, United States college from two consecutive annual student cohorts participated in an online survey (77 women, 69 men, 15 refused to answer). The sample was 83.2% Caucasian, 3.1% African American or Black, 2.5% Asian or Asian-American, and 11.3% other ethnicity. Participants did not record their specific age, but all indicated that they graduated in the late 1980s. The lead author entered participants into a raffle to win one of three $50 gift certificates to the university’s online bookstore.

#### Procedure and Measures

Participants completed an online survey through SurveyMonkey. They responded to the measures below and offered the opportunity to enter their email (separately from their data) for the gift card raffle.

### University Nostalgia

The measure of university nostalgia was the same as in Study 1, except for the name of the alma mater (now “X College”; 1 = *not at all*, 7 = *very much*). Cronbach’s alpha was 0.83.

### Belonging With University Community

We measured belonging with the university community using the same 6-item composite scale as in Study 1 (1 = *not at all*, 7 = *very much*). Also as in Study 1, the social connectedness and group identification scales were highly correlated [*r*(159) = 0.78]. Cronbach’s alpha was 0.92.

### Self-Continuity

We measured self-continuity with the 4-item scale of Study 1 (1 = *not at all*, 7 = *very much*). Cronbach’s alpha was 0.88.

### Meaning in Life

We measured meaning in life with an adapted version of the Meaning in Life Questionnaire (Steger et al., 2006). We shortened the original 10-item scale to eight items focusing on felt presence of meaning in life. Sample items include: “I feel life has a purpose when I think of X College or am at X College” and “I feel a sense of meaning when X College comes to mind” (1 = *absolutely untrue*, 7 = *true*). Cronbach’s alpha was 0.95.

### Past University Experiences

Participants reported how positive their university experiences were on a 4-item scale that we created for the purpose of this study (1 = *not at all*, 7 = *completely*). Sample items include: “How positive overall emotionally was your X College experience?” and “To what extent did you feel that you weren’t fully accepted at X College?” (reverse-scored). Cronbach’s alpha was 0.77.

### Subjective Well-Being

We measured subjective well-being with the Subjective Vitality Scale (Ryan and Frederick, 1997). This 7-item scale assessed the extent to which participants felt full of energy and alive. Sample items include: “I look forward to each new day” and “I have energy and spirit” (1 = *not at all*, 7 = *completely*). Cronbach’s alpha was 0.90.

### Socializing With Other Alumni

Participants identified their interest in socializing with other alumni by responding to the item: “How much do you plan to socialize with fellow X College alums in the coming year?” (1 = *not at all*, 7 = *a great deal*).

### Informal Visits to Campus

Participants also indicated how often they visited campus by responding to the item: ‘‘How many times have you visited College X informally (not a reunion) since your graduation?’’ In particular, they were instructed to enter a number no less than 0.^1^

Volunteering. Participants responded to the question “How much time do you plan to donate to the College in the form of serving the College or your class, service projects, etc., in the next year?” (1 = *not at all*, 7 = *a great deal*).

### Reunion Interest

We measured reunion attendance and interest with two items reflecting past attendance and future interest in class reunions. These were: “How much interest do you have in attending the next reunion?” and “How many official reunions have you attended at College X?” (1 = *not at all*, 7 = *a great deal*). (Given that we recruited all participants from the same two adjacent class years, we did not need to ask about the overall number of reunions held by participants’ university class).

### Charitable Donations

Participants responded to several measures relating to charitable donations in the form of numeric amounts in United States dollars. These included: amount they intended to donate that year (“How much money do you plan to give to College X this year?”); average donation amount (“What was your average monetary gift to College X since you graduated?”); and largest donation amount (“What was your largest gift to College X since you graduated?”).

### Demographics

Participants reported their gender, ethnicity, and class year. Due to an oversight, we did not include income in the demographics section.

### Results

#### Analysis Strategy

Four variables (average gift, largest gift, planned donation amount, number of visits) were highly right-skewed and leptokurtic. We log-transformed them, thus meeting assumptions for linear regression (skew/kurtosis < 1.5). We display means, medians, and standard deviations of all variables in Table 4, and correlations in Table 5. As in Study 1, connectedness and identification with the university community evidenced multicollinearity [reminder: *r*(159) = 0.78], so we averaged the six items across the connectedness and identification measures into a composite reflecting university belongingness. The Cronbach’s alpha for university belongingness was 0.92.

**TABLE 4:** Means, standard deviation, and median of study 2 variables.

Variable	Mean	SD	Median
University Nostalgia	4.14	1.12	4.29
Connectedness to university community	4.36	1.50	4.57
Identification with university community	5.22	1.43	5.67
Belongingness with the university community	4.94	1.46	5.17
Self-continuity	4.80	1.35	4.75
Felt meaning in life	4.43	1.46	4.50
Positive university experiences	5.17	1.12	5.50
Well-being	5.07	0.92	5.29
Past socializing with alumni	3.87	1.55	4.00
Planned future socializing with alumni	4.09	1.65	4.00
Planned volunteering (hours)	3.17	1.93	3.00
Past university visits (number of visits)	29.51	140.30	5.00
Class reunions attended (number of reunions)	2.60	1.78	3.00
Interest in attending upcoming reunion	4.55	1.69	5.00
Years donated (number of years)	16.63	8.93	20.00
Average gift amount (dollars)	$809	$2,445	$100
Largest gift amount (dollars)	$6,007	$23,642	$500
Planned future donation amount (dollars)	$2,318	$9,576	$125

*Median income = $100,000. All variables without a specified rate are measured on a 1 *(low*) to 7 (*high*) rating scale.*

**TABLE 5:** Bivariate correlations among study 2 variables (*N* = 161).

Variables	1.	2.	3.	4.	5.	6.	7.	8.	9.	10.	11.	12.	13.	14.	15.	16.	17.	18.	19.
1. Univ. Nostalgia	–																		
2. Connection	0.65***	–																	
3. Identification	0.59***	0.78***	–																
4. Belonging	0.69***	0.94***	0.87***	–															
5. Meaning in life	0.62***	0.57***	0.69***	0.68***	–														
6. Self-continuity	0.47***	0.61***	0.62***	0.66***	0.57***	–													
7. Positive univ. experiences	0.41***	0.44***	0.50***	0.52***	0.40***	0.30***	–												
8. Well-being	0.02	0.23**	0.16*	0.22**	0.12	0.13	0.07	–											
9. Past socialization	0.43***	0.59***	0.29***	0.49***	0.28***	0.39***	0.25**	0.21**	–										
10. Planned socialization	0.49***	0.68***	0.42***	0.62***	0.39***	0.45***	0.32***	0.25**	0.90***	–									
11. Willingness to volunteer	0.55***	0.66***	0.51***	0.65***	0.48***	0.46***	0.26***	0.22**	0.42***	0.49***	–								
12. Past reunion attend.	0.38***	0.48***	0.36***	0.46***	0.26**	0.25**	0.32***	0.11	0.37***	0.36***	0.47***	–							
13. Planned reunion attend.	0.54***	0.64***	0.45***	0.62***	0.43***	0.42***	0.49***	0.18*	0.43***	0.49***	0.54***	0.59***	–						
14. Informal campus visits	0.13	0.22**	0.12	0.20*	0.15	0.09	–0.10	0.06	0.13	0.14	0.15	0.10	0.06	–					
15. Past years donated	0.36***	0.55***	0.45***	0.57***	0.36***	0.24**	0.43***	0.22**	0.19*	0.27***	0.41***	0.52***	0.45***	0.19*	–				
16. Past average gift amount^†^	0.19*	0.25**	0.10	0.21*	0.22**	0.16	–0.11	0.20*	0.10	0.11	0.29***	0.13	0.07	0.20*	0.20*	–			
17. Past largest gift amount^†^	0.19*	0.20*	0.17*	0.20*	0.20*	0.15	0.01	0.13	0.13	0.14	0.23**	0.20*	0.14	0.05	0.17*	0.82***	–		
18. Future willing to donate	0.40***	0.52***	0.42***	0.53***	0.43***	0.28***	0.34***	0.18*	0.18*	0.31***	0.39***	0.41***	0.48***	0.16*	0.67***	0.14	0.11	–	
19. Future willing donation amount^†^	0.19*	0.19*	0.13	0.18*	0.21**	0.13	–0.05	0.14	0.12	0.14	0.22**	0.17*	0.12	0.08	0.16*	0.80***	0.90***	0.09	–

*p < 0.05, **p < 0.01, ***p < 0.001, and † variable log-transformed due to strong positive skewness.Univ, university; Attend., attendance.

We first conducted hierarchical regression analyses to identify the relation between university nostalgia and participants’ intentions to engage with their alma mater. We did not control for graduation year in these models, as we recruited participants from the same class cohort. Given that current engagement and positive thoughts and feelings could be explained by having had a positive experience at university, we controlled for positive past university experience in all models, and past engagement in all models except volunteer plans^2^ in step 1 of each regression model. In step 2 of each model, we entered university nostalgia. We present results of these analyses in Table 6.

**TABLE 6:** Regression models for study 2 well-being and alumni engagement outcomes.

Dependent variable (continuous)	Predictor	Step 1						Step 2					
		β	*p(*β*)*	*F*	*df*	*p*(*F*)	*R^2^_*adj*_*	β	*p(*β*)*	*F*	*df*	*p*(*F*)	*R^2^_*adj*_*
Subjective well-being	Positive college experiences University Nostalgia	0.08	0.351	0.88	1, 157	0.348	0.01	0.09 −0.04	0.313 0.690	0.52	2, 156	0.597	0.01
Socializing with other alumni	Positive college experiences Past socialization University Nostalgia	0.11 0.87	0.004 <0.001	333.27	2, 155	<0.001	0.81	0.07 0.84 0.10	0.055 <0.001 0.019	230.70	3, 154	<0.001	0.82
Volunteering (overall amount)	Positive college experiences University Nostalgia	0.26	0.001	11.76	1, 157	0.001	0.06	0.05 0.53	0.534 <0.001	34.00	2, 156	<0.001	0.30
Reunion interest	Positive college experiences Past reunion attendance University Nostalgia	0.34 0.48	<0.001 <0.001	63.44	2, 155	<0.001	0.44	0.24 0.40 0.30	<0.001 <0.001 <0.001	54.80	3, 154	<0.001	0.51
Informal visits	Positive college experiences Past reunion attendance University Nostalgia	−0.05 0.23	0.542 0.008	3.69	2, 146	0.03	0.04	−0.12 0.17 0.23	0.172 0.061 0.014	4.59	3, 145	0.004	0.09
Donation (amount)	Positive college experiences Past donation average amount University Nostalgia	0.12 0.81	0.025 <0.001	147.52	2, 120	<0.001	0.70	0.08 0.77 0.12	0.150 <0.001 0.045	102.28	3, 122	<0.001	0.71

		***OR***	***p(OR)***	**Model χ2**	***Df***	***p*(χ2)**	***Nagelkerke R*^2^**	***OR***	***p(OR)***	**Model χ2**	***df***	***p*(χ2)**	***Nagelkerke R*^2^**

Donation (y/n)	Positive college experiences Past donation frequency University Nostalgia	1.49 1.51	0.273 <0.001	82.15	2	<0.001	0.72	1.06 1.50 1.16	0.888 <0.001 0.032	88.26	3	<0.001	0.76

We then examined three potential mediators to explain the links of university nostalgia with subjective well-being and engagement outcomes above and beyond the covariates: belonging with the university community, self-continuity, and meaning in life. To test these mediators, we used Hayes’s (2018) PROCESS macro v3.4 in SPSS with 5,000 bootstrapped iterations. For each outcome, we report both simple and parallel mediation analyses, as in Study 1 (PROCESS model 4). We controlled for positivity of university experiences and, when applicable, past engagement. We included positive college experiences and prior engagement in the model of the dependent variable, but not in the model of the mediator as in Study 1.

#### Subjective Well-Being

A hierarchical regression modeled the relation between university nostalgia and current subjective well-being, controlling for positivity of university experiences (Table 6). University nostalgia did not significantly predict subjective well-being (β = −0.04, *p* = 0.694, *R*^2^Δ = 0.001).

Although the total ‘‘effect’’ of university nostalgia on subjective well-being was not significant, we probed whether there were significant indirect effects of university nostalgia on subjective well-being through the three mediators^3^. When assessing each mediator separately, the relation between university nostalgia and subjective well-being was significantly mediated by university belonging [*ab* = 0.24, 95% CI (0.13,0.36)], but not self-continuity [*ab* = 0.06, 95% CI (−0.002,0.14)] or meaning in life [*ab* = 0.10, 95% CI (−0.03,0.22)]. In the parallel mediation analysis, university belonging uniquely mediated the relation between nostalgia and subjective well-being [*ab* = 0.25, 95% CI (0.10,0.40)]. However, self-continuity [*ab* = −0.01, 95% CI (−0.12,0.08)] and meaning in life [*ab* = 0.02, 95% CI (−0.12,0.15)] did not. Taken together, university nostalgia predicted increased subjective well-being *via* university belonging.

#### Socializing With Other Alumni

University nostalgia predicted significantly greater interest in socializing with other alumni (β = 0.10, *p* = 0.009, *R*^2^Δ = 0.01). When assessing each potential mediator separately, the relation between university nostalgia and interest in socializing with other alumni was significantly mediated by university belonging [*ab* = 0.17, 95% CI (0.09,0.26)] and meaning in life [*ab* = 0.07, 95% CI (01,0.15)]. However, self-continuity was not a significant mediator [*ab* = 0.03, 95% CI (−0.02,0.08)]. In the parallel mediation analysis, university belonging remained the only significant mediator [*ab* = 0.35, 95% CI (0.21,0.52)]. Self-continuity [*ab* = 0.02, 95% CI (−0.06,0.09)] and meaning in life [*ab* = 0.01, 95% CI (−0.09,0.12)] were not significant mediators.

#### Volunteering

University nostalgia predicted increased planned volunteering (β = 0.53, *p* < 0.001, *R*^2^Δ = 0.24). In separate mediation analyses, this relation was significantly mediated by university belonging [*ab* = 0.38, 95% CI (0.27,0.51)], self-continuity [*ab* = 0.12, 95%CI (0.05,0.20)], and meaning in life [*ab* = 0.15, 95% CI (0.04,0.25)]. In the parallel mediation analysis, university belonging remained the only significant mediator (*ab* = 0.31, 95% CI [0.20,0.45]). Self-continuity [*ab* = 0.01, 95% CI (= −0.07,0.07)] and meaning in life [*ab* = 0.01, 95% CI (−0.08,0.10)] were not significant mediators.

#### Reunion Interest

University nostalgia predicted greater interest in attending the next reunion (β = 0.30, *p* < 0.001, *R*^2^Δ = 0.07). In separate mediation analyses, the relation between university nostalgia and participants’ interest in attending the upcoming reunion was significantly mediated by university belonging [*ab* = 0.17, 95% CI (0.04,0.31)] and self-continuity [*ab* = 0.06, 95% CI (0.002,0.15)], but not by meaning in life [*ab* = 0.06, 95% CI (−0.03,0.16)]. In the parallel mediation analysis, none of the three indirect effects were significant [university belonging, *ab* = 0.15, 95% CI (−0.02,0.32); self-continuity, *ab* = 0.03, 95% CI (= −0.04,0.11); meaning in life, *ab* = −0.01, 95% CI (−0.10,0.09)].

#### Informal Visits to Campus

University nostalgia significantly predicted greater number of visits to campus outside of official reunions (β = 0.23, *p* = 0.014, *R*^2^Δ = 0.05). When assessing mediators separately, this relation was significantly mediated by university belonging [*ab* = 0.26, 95% CI (0.01,0.45)]. Self-continuity [*ab* = 0.03, 95% CI (−0.06,0.12)], and meaning in life [*ab* = 0.10, 95% CI (−0.06,0.23)] did not significantly mediate the relation. In the parallel mediation analysis, none of the indirect effects were significant [university belonging, *ab* = 0.30, 95% CI (−0.01,0.55); self-continuity, *ab* = −0.06, 95% CI (= −0.16,0.04); meaning in life *ab* = 0.03, 95% CI (−0.13,0.17)].

#### Charitable Donations

We first conducted a logistic hierarchical regression to model the likelihood that participants planned to donate (vs. not donate) to their alma mater as a function of their level of university nostalgia. University nostalgia predicted greater odds of a planned donation (OR = 1.16, *p* = 0.032, *R*^2^Δ = 0.04). We then conducted a hierarchical OLS regression analysis to assess whether university nostalgia predicted the amount that participants were willing to donate. We log-transformed the variable for planned donation amount in order to address strong positive skewness in the original variable. University nostalgia predicted higher planned donation amounts (β = 0.12, *p* = 0.032, *R*^2^Δ = 0.01).

We first assessed charitable donations as a dichotomous outcome in a series of simple logistic mediation analyses. The relation between university nostalgia and participants’ likelihood of intending to donate (vs. not donate) to the alma mater, calculated as an odds ratio, was significantly mediated by university belonging [*ab* = 1.81, 95% CI (0.44, 4.09)]. Self-continuity [*ab* = 1.15, 95% CI (0.76, 5.29)] and meaning in life [*ab* = 1.74, 95% CI (0.86, 5.74)] did not significantly mediate the relation. In the parallel mediation analysis, none of the indirect effects were significant [university belonging, *ab* = 1.68, 95% CI (0.77, 16.39); self-continuity, *ab* = 0.88, 95% CI (= 0.26, 1.93); meaning in life, *ab* = 1.67, 95% CI (0.67, 27.24)].

We next assessed charitable donations as the total amount that participants reported planning to donate to the alma mater (log-transformed). When assessing mediators separately, the relation between university nostalgia and planned donation amount was significantly mediated by university belonging [*ab* = 0.10, 95% CI (0.002,0.21)]. Self-continuity [*ab* = 0.03, 95% CI (−0.02,0.09)] and meaning in life [*ab* = 0.08, 95% CI (−0.001,0.17)] did not significantly mediate the relation. In the parallel mediation analysis, none of indirect effects were significant [university belonging, *ab* = 0.06, 95% CI (−0.06,0.19); self-continuity, *ab* = 0.001, 95% CI (= −0.08,0.06); meaning in life, *ab* = 0.06, 95% CI (−0.04,0.16)].

#### Moderation by Positivity of University Experience

Finally, we examined whether positivity of past university experiences moderated the links of university nostalgia with subjective well-being and university engagement. Results revealed evidence for a moderating role of past university experiences pertaining to two indices of university engagement: planned donation amounts and intentions to attend an upcoming reunion (for all other outcome variables, University Nostalgia × Past University Experience interaction *p*s > 0.13).

**FIGURE 1:**
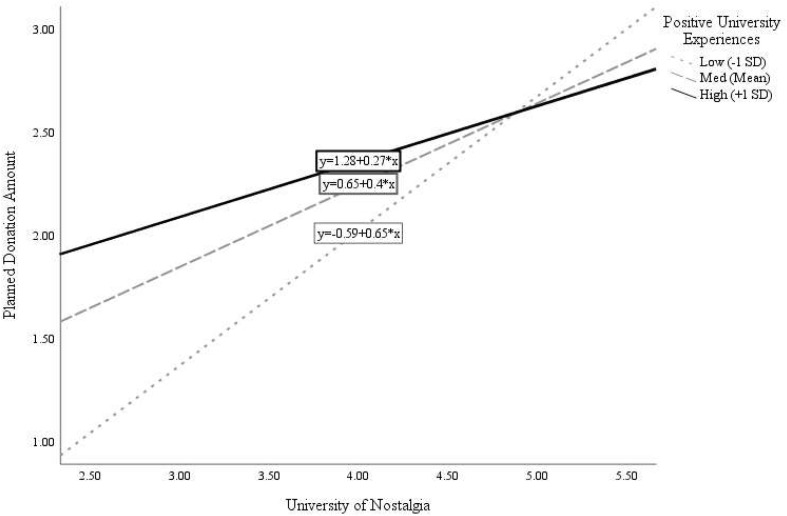
Moderation of relation between University Nostalgia and charitable donations positivity of University experiences.

**FIGURE 2:**
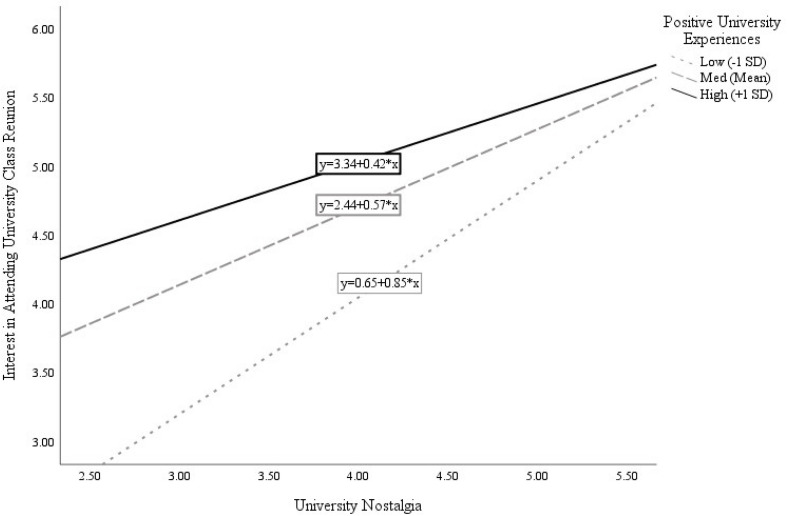
Moderation of relation between University Nostalgia and reunion interest by positive University experiences.

Participants’ positive experiences in university significantly moderated the association of nostalgia with planned donation amounts [interaction β = −0.17, *F*(1, 124) = 4.90, *R*^2^Δ due to interaction = 0.03, *p* = 0.029]. Analysis of the simple slopes (Figure 1) indicates that the relation between nostalgia and donation amount was stronger for participants who scored low (−1 *SD*) on positive past university experiences (β = 0.64, *p* < 0.001) than for those who scored high (+1 *SD*) on positive past university experiences (β = 0.26, *p* = 0.031). The relation between university nostalgia and participants’ plans to attend the upcoming reunion was also moderated by positivity of university experiences (interaction β = −0.19, *R*^2^Δ due to interaction = 0.02, *p* = 0.024). As shown in Figure 2, the positive relation between university nostalgia and reunion interest was again more pronounced for participants who scored low (−1 *SD*) on positive university experiences (β = 0.85, *p* < 0.001) than those who scored high (+1 *SD*) on positive university experiences (β = 0.42, *p* = 0.001).

These findings offer tentative support for the idea that, for individuals who had a relatively negative (compared to positive) overall university experience, the few nostalgic memories that they do cherish assuage their overall negative experience, thereby protecting and sustaining some aspects of university engagement.

### Discussion

Study 2 replicated and extended the findings of Study 1. Consistent with Hypothesis 1, university nostalgia predicted willingness to engage with the university in several ways: intentions to donate time volunteering and donate money, plans to spend more time socializing with fellow alumni, attend an upcoming class reunion, and visit campus (a new outcome in Study 2). Stated otherwise, university nostalgia predicted outcomes at both the relational (e.g., time with fellow alumni) and collective (e.g., volunteering for the university) levels. Importantly, and consistent with Study 1, these findings held while controlling for past engagement and positivity of past university experiences (a new control variable in Study 2). University nostalgia was not directly associated with subjective well-being (another new outcome in Study 2), but was linked to it indirectly, *via* increased university belonging.

The mediational findings for Study 2 generally were consistent with those of Study 1. In simple mediation analyses, university belonging was a significant mediator of the relation between university nostalgia and subjective well-being, as well as all six engagement outcomes. Both self-continuity (volunteering and reunion interest) and meaning in life (socializing with other alumni and volunteering) mediated the link between university nostalgia and two engagement outcomes.

In parallel mediation analyses, university belonging uniquely mediated the link between university nostalgia and three outcomes. No other unique indirect effects emerged. The generally weaker results in the parallel mediation analyses are likely due to shared variance among the predictors. Indeed, for three engagement outcomes (i.e., reunion interest, campus visits, planned donation amount), the parallel mediation analysis revealed no significant indirect effects, yet the total, combined indirect effect of all three mediators was significant [reunion interest, *ab* = 0.17, 95% CI (= 0.02,0.32); campus visits, *ab* = 0.28, 95% CI (0.01,0.48); planned donation amount, *ab* = 0.12, 95% CI (= 0.01,0.24)]. Together, these findings provide further qualified support for Hypothesis 2, in particular as it relates to the mediating role of university belonging.

Positivity of experience moderated the relation of university nostalgia with two engagement outcomes in a manner that helps answer the question of who benefits from feeling nostalgic. Whereas it may seem plausible that nostalgia would mostly benefit individuals who have a large reservoir of positive past experiences to look back on, our findings suggest otherwise. University nostalgia was more positively associated with planned donation amounts and reunion attendance intentions among participants who reported more negative overall university experiences. That is, those who had the least positive university experiences benefited the most from university nostalgia. We hasten to add that, although donating and attending reunions arguably are the two most salient examples of university engagement, the effect did not extend to all outcome variables. Regardless, future research would need to test the replicability of our novel finding and also examine more closely this link. Perhaps the negative university experiences lose their potency when examined through the rose-colored glasses of nostalgia, or greater university nostalgia directly changes expectations about future university engagement.

## General Discussion

In his *Requiem for a Nun*, (Faulkner, 1951, p.73) wrote: “The past isn’t dead. It isn’t even past.” Many university alumni never really leave their alma mater. They take it with them, having incorporated in themselves close relationships, university values, and cherished memories. Rather than being a closed chapter in their lives, they reflect on those formative years, and this university nostalgia continues to influence them. In two studies, participants’ university nostalgia was associated with intentions to engage with their alma mater and socialize with fellow alumni. Study 1 involved graduates (aged 18–79 years) from a large public university in the southern United States, and Study 2 involved graduates of a private northeast university from two consecutive classes in their 40s. In both studies, university nostalgia was positively associated with greater willingness to donate money to the alma mater and in higher amounts, volunteer for the alma mater, socialize with fellow alumni/ae, and plan to attend upcoming university reunions. University nostalgia predict engagement above and beyond past engagement (both studies) and when controlling for the positivity of past university experiences (Study 2).

### Mediation by University Belonging

The relation between university nostalgia and university engagement outcomes was mediated by university belonging: connectedness to, and identification with, the university community. In Study 1, university belonging mediated all but one of the links (decision to donate) in single mediation analyses. When examined in parallel with the other mediator of self-continuity, university belonging uniquely mediated the links between university nostalgia and socializing with fellow alumni as well as the intent to volunteer. The mediation results for Study 2 were even clearer. In simple mediation analyses, university belonging mediated the links of university nostalgia with subjective well-being and all six engagement variables. In parallel mediation analyses (with self-continuity and meaning in life), university belonging continued to mediate the associations of university nostalgia with subjective well-being and socializing.

The mediational analyses provided strong evidence for the role of university belonging. This composite measure was an amalgam of university connectedness and university identification due to their high correlation. The belongingness measure has been used and validated in prior nostalgia research (Wildschut et al., 2006; Hepper et al., 2012a), and the identification measure was adapted from previous work (Tarrant et al., 2004). However, we chose a subset (three items) from each scale. It is possible that our shorter scales resulted in higher correlations. It is also possible that belonging and identification simply are more highly correlated for university nostalgia; future research should address this issue. Future work also should assess the mediational potency of meaning in life and self-continuity with different measures and samples. Further, mediational analyses do not speak to causality, but experiments (e.g., manipulating university belonging) and longitudinal studies may provide additional support for our findings.

Moreover, longitudinal studies should go beyond measuring behavioral intentions and assess university-directed behavior, such as hours volunteered, actual reunion attendance, or money donated. Due to the self-report nature of this work and the potential social desirability demands of requests to donate or volunteer, participants’ self-reported intentions are likely to overestimate their actual readiness to sacrifice money or time for the alma mater (Ajzen et al., 2004). However, given converging evidence that nostalgia does motivate actual giving and helping behavior (e.g., Zhou et al., 2008; Juhl et al., 2020a, b), we expect that the trajectory of our findings would likely be replicated in future studies of *in vivo* behavior.

Taken together, these mediation findings suggest that the sense of belonging to one’s university is a key mechanism through which university nostalgia influences activities directed toward one’s alma mater as well as fellow alumni. Although these findings are correlational, past experimental research supports a causal path from nostalgia to social connectedness and identification (Wildschut et al., 2014), and from social connectedness and identification to tangible actions to benefit the group (Tyler and Blader, 2003). Notwithstanding, some of these associations may be bidirectional. Repeated university engagement, such as spending time with fellow university alumni or attending reunions, may in turn heighten university nostalgia. Attending a reunion or spending time with university friends may increase the frequency of nostalgic reverie as well as augment social connectedness and identification with the university. These regular injections of university nostalgia *via* university engagement cascade into a feedback loop in which university nostalgia and engagement increase over time. Future work, particularly experimental or longitudinal, may clarify this issue.

### Moderation by Positivity of Past Experience

Research has explored the boundary conditions of nostalgia’s benefits from several angles. An individual difference approach has found that nostalgia’s benefits typically extend widely. For example, a recent well-powered meta-analysis revealed that neuroticism does not moderate the benefits of induced nostalgia (Frankenbach et al., 2020). We addressed this issue by asking whether people who report a negative past university experience can derive benefits from university nostalgia. It may seem plausible that having fewer positive memories to draw upon might prevent an individual from experiencing some of the social or existential benefits of nostalgia. We found the opposite. For two engagement outcomes—planned financial donation amount and upcoming reunion plans—the link with university nostalgia was strongest among those alums who had the most negative university experiences. This surprising finding demands replication, but it may reflect the capacity for collective nostalgia to serve as a psychological resource that enhances willingness to socialize with others and maintain loyalty with ingroups (Sedikides and Wildschut, 2019). Future research should examine more closely the possible role of nostalgia in forgiveness and repairing social bonds. It is possible, for example, that dispositional nostalgia for a group, such as one’s university, promotes forgiveness of perceived offenses through greater willingness to empathize (Juhl et al., 2020b) or engage in recollection that might be painful (Batcho, 2013) of past offenses by individuals within the university. This finding resonates with the idea, voiced so elegantly by Dostoyevsky (2007) in *The Brothers Karamazov*, that “… if one has only one good memory left in one’s heart, even that may sometime be the means of saving us” (p. 868).

### Additional Limitations and Future Directions

In addition to the aforementioned limitations (e.g., the university belonging mediator, measuring behavioral intentions), our samples had some potential weaknesses. Although the two samples were reasonably diverse on characteristics such as age as well as university region and type (i.e., one smaller private school in the northeast and one large public school in the south), both were United States universities. Future research could examine how both university culture and the larger culture might moderate these findings. Relatedly, cross cultural work could attempt to examine the characteristics of college that afford the greatest wellsprings of nostalgic reverie. We suspect elements that foster social connection (e.g., dormitory or apartment life and college clubs) and social identity (e.g., college sports) are the most promising.

### Coda

Nostalgia for one’s university days motivates graduates to stay connected to their university community by volunteering and donating money, as well as stay connected to their fellow graduates by socializing with them. Feelings of university belonging explain most of these links. These findings are captured by the alma mater of the United States college from which we recruited in Study 2:

*Though ‘round the girdled earth they roam, her spell on them remains*…
Around the world they keep for her their old undying faith.

## Data Availability Statement

The raw data supporting the conclusions of this article will be made available by the authors, without undue reservation.

## Ethics Statement

The studies involving human participants were reviewed and approved by Virginia Commonwealth University Institutional Review Board. The patients/participants provided their written informed consent to participate in this study.

## Author Contributions

All authors listed have made a substantial, direct and intellectual contribution to the work, and approved it for publication.

## Conflict of Interest

The authors declare that the research was conducted in the absence of any commercial or financial relationships that could be construed as a potential conflict of interest.

## Footnotes

1 Responses ranged from 0 to 1,000, with three respondents reporting greater than 100 visits. We winsorized these three participants to create a “100 +” interval bin in order to reduce right skewness in the variable.

2 Due to an error in the Study 2 survey construction, we asked participants only about future volunteerism plans and not about past volunteerism frequency.

3 See Hayes (2018) for a succinct explanation for why the absence of a significant total X-Y relation does not necessarily preclude a logically sound indirect X-Y effect through one or more mediators.
